# Preliminary Experiment on the Effect of 18% Substitute Salt on Home Blood Pressure Variability in Hypertensives

**DOI:** 10.1155/2021/9993328

**Published:** 2021-09-02

**Authors:** Jing Li, Lisha Mu, Huakun Rao, Yangfeng Wu, Hao Wang, Hongbing Tao, Lihong Mu

**Affiliations:** ^1^Tongji Medical College of Huazhong University of Science and Technology School, Wuhan, China; ^2^School of Public Health and Management, Research Center for Medicine and Social Development, Innovation Center for Social Risk Governance in Health, Chongqing Medical University, Chongqing, China; ^3^Peking University School of Public Health and Clinical Research Institute, Beijing, China; ^4^Chongqing Nan'an District People's Hospital, Chongqing, China

## Abstract

At present, the effect of substitute salt in reducing sodium intake and blood pressure is relatively clear. The present study is a phase I clinical trial involving 43 hypertensives in which the effect of 18% sodium substitute salt on the home blood pressure variability (BPV) was observed for 8 weeks with weekly follow-up. Finally, 4 patients were lost, and 39 patients completed the intervention and were included in the analysis. Daily home blood pressure and weekly adverse events were collected. The systolic blood pressure (SBP) in the morning (−10.0 mmHg, 95% CI: −16.5 to −3.5, *P* = 0.003), SBP at night (−10.2 mmHg, 95% CI: −16.1 to −4.3, *P* = 0.001), and diastolic blood pressure (DBP) at night (−4.0 mmHg, 95% CI: −7.1 to −0.8, *P* = 0.014) decreased significantly. Also, there was no statistically significant change in morning (*F* = 1.137, *P* = 0.352) and night diastolic (*F* = 0.344, *P* = 0.481) BPV and morning systolic BPV (*F* = 0.663, *P* = 0.930) over time during the intervention period, except for that night systolic BPV had a downward trend (*F* = 2.778, *P* = 0.016) and had decreased 2.04 mmHg (95% CI: 0.84 to 3.23, *P* = 0.001) after intervention. The use of 18% of the substitute salt did not increase BPV during the intervention and even may decrease it, which indicates its control effects on blood pressure. This study is the first one to observe the effect of 18% sodium substitute salt on the home blood pressure variability, providing a basis for further experiments.

## 1. Introduction

It is well known that excessive salt intake is closely related to an increase in blood pressure and the risk of cardiovascular-related events [1]. The World Health Organization recommends that the daily salt intake per person should be less than 5 g per day, while current salt intake is about 12 g or more in many countries, including China [2]. Therefore, it is imperative to find an effective salt restriction measure. Studies have shown that substitute salt is a more cost-effective measure to restrict salt intake [3, 4]. Research showed that substitute salt can lower the blood pressure (−7.52/−4.25 mmHg) compared to common salt [5–9]. Spontaneous variation in blood pressure is referred to as BPV due to the effects of day and night changes, weather changes, and other factors [10, 11]. A large number of studies [12–14] have confirmed that blood pressure variability is closely related to the risk of future cardiovascular events in patients with hypertension and is an independent predictor of blood pressure mean, which has an important prognostic value for patients with hypertension [15]. However, there has been little research on the effect of substitute salt on BPV. This study aimed to investigate whether the intake of alternative salts would alter BPV in patients with hypertension.

## 2. Methods

### 2.1. Participants

This study was completed in the Public Health Department of the People's Hospital of Nan'an District, Chongqing, China. A total of 43 patients diagnosed with primary hypertension were included from May to July 2018. Inclusion criteria: (1) age in the range of ≥50 and ≤ 75 years; (2) not having plans to move out of the community in the next three months; (3) not cooking at home less than 3 times or one day during the study; and (4) written informed consent provided before enrollment in the trial. Exclusion criteria: (1) history of acute myocardial infarction or stroke in the past 3 months and history of malignancy or expected lifetime less than 1 year; (2) hypercortisolism or aldosteronism; (3) acute disease, such as upper respiratory infection, fever, and diarrhea; (4) disease or disabilities that could exert potential influence on their adherence to the intervention, including deafness and dementia, as well as severe depression and other mental disorders; (5) salt substitute use in the family; (6) family members not willing to use the salt substitute; (7) chronic renal failure at stage 4 or above or with renal replacement therapy; (8) abnormal liver function, with alanine aminotransferase (ALT) or aspartate aminotransferase (AST) level greater than 2 times the upper limit of normal, or total bilirubin level greater than the upper limit of normal; and (9) abnormal blood potassium level, <3.5 mmol/L or >5.5 mmol/L, or current use of potassium-preserving diuretics. The study has been approved by the Institutional Review Board of Peking University (IRB00001052−17110). The trial was registered at http://www.clinicaltrials.gov (NCT03226327).

### 2.2. Study Design and Intervention

This study is a phase I clinical trial of single arm. After the subjects and their family signed the informed consent, subjects completed baseline questionnaires and medical examination (including general physical examination, blood pressure, and 24-hour urine test). 18% substitute salt instead of traditional salt was used for 8 weeks, and patients were measured home blood pressure every day, while follow-up staff visited the patients once a week to collect information of antihypertensive drug use and safety.

### 2.3. Materials and Instruments

In this study, the 18% sodium substitute salt of “Man Li Kang” was developed by Chongqing Institute of Biotechnology Co., Ltd.: name: solid compound condiment, standard of execution:Q/SWS0025S, food production license number: SC10650012000709, and food circulation permit: SP5009051610016538, and the main ingredients include potassium chloride (35%), sodium chloride(18%), and calcium chloride(10%). The blood pressure measuring instrument adopts a pulse-wave electronic sphygmomanometer (RBP-9801).

### 2.4. Blood Pressure Measurement

Before the start of the study, follow-up staff was trained in relevant knowledge of blood pressure measurement. Then, each subject was trained in blood pressure measurement by the follow-up staff, and a home blood pressure measurement handbook was distributed to all subjects. Patients were required to complete 3 blood pressure measurements at time in the morning and evening (from 6 to 9 o'clock am/pm) every day. Besides, during the weekly follow-up, the follow-up staff would confirm with the subjects whether the blood pressure measurement method is correct or not. Also, the blood pressure measurement results were automatically uploaded to the terminal management system in real time through the device. The researchers collected the blood pressure data for 8 weeks for sorting and analysis.

### 2.5. Substitute Salt Weighing and 24-Hour Urine Test

Follow-up staff used a uniform electronic scale to weigh the weekly salt used by each patient and calculate the weekly salt usage of the patient.

The 24-hour urine of patients was collected at baseline and after the intervention, sent to a local grade-A tertiary hospital for testing. The items include 24-hour urinary sodium, potassium, calcium, magnesium, urinary creatinine (Cr), and urinary microalbumin (U-ALB).

### 2.6. Statistical Analysis

Three blood pressure measurements were required per time, and the last 2 averages were included in the analysis. BPV is represented by standard deviation (SD) of blood pressure per week. The baseline blood pressure was taken as an average measured three times, respectively, in the morning and evening on the first day of intervention.

Statistical analysis was carried out using SPSS 22.0 (IBM, Armonk, NY, USA). A total of 1953 pieces of complete blood pressure measurement data were collected in the morning, with a missing rate of 9.45% and 1871 in the evening, with a missing rate of 14.33%. The missing data of home blood pressure were filled by multiple imputations [16]. Quantitative normal distribution data are described by mean and standard deviation, and qualitative data are described by frequency. Urine sodium and potassium before and after intervention were compared using the paired T, and the linear mixed models were used to analyze the change of blood pressure and its variability. *P* values less than 0.05 were considered statistically significant.

## 3. Results

### 3.1. Baseline

In this study, we enrolled 43 patients, with 4 lost because of the intolerability of the taste of the substitute salt. 39 patients were finally analyzed by per protocol (PP), including 20 males and 19 females, aged 67.1 ± 7.9 years, with a body mass index (BMI) of 26.6 ± 3.6. Among them, 14 subjects reduced the doses of antihypertensive drugs and 25 subjects had the doses unchanged. Blood pressure at baseline is shown in Table 1.

### 3.2. Use of 18% Substitute Salt and the 24-Hour Urine Test

**Figure 1:**
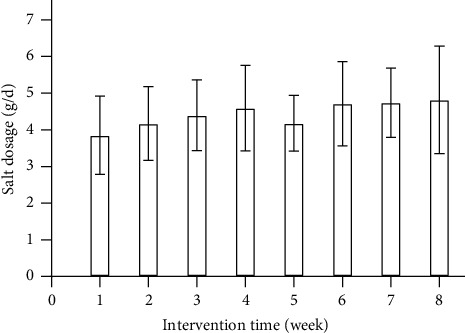
Per capita daily 18% substitute salt dosage (*n* = 39).

After 8 weeks of intervention, the urine potassium increased by 8.79 mmol/24 h on average (*P* = 0.021), urinary sodium/potassium decreased significantly compared with baseline (*P* = 0.001), and urine sodium decreased by 13.82 mmol/24 h on average, but there was no statistically significant difference (*P* = 0.220). U-ALB/Cr increased compared with baseline (*P* = 0.002), but urine calcium and magnesium had no significant changes compared with baseline (*P* > 0.05) (Table 2). The per capita salt of the subjects weighed by the follow-up staff each week was around 3–5 g/d (Figure 1).

### 3.3. Blood Pressure and Its Variability

**Figure 2:**
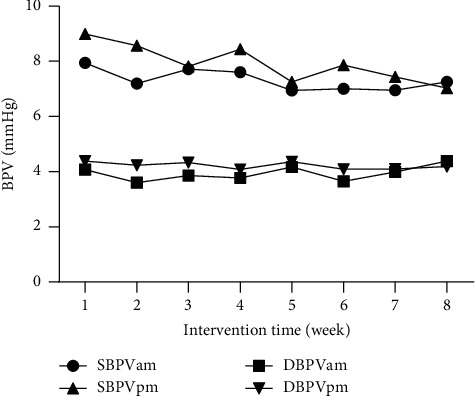
Weekly BPV change trend of patients.

The analysis of the mixed linear model showed that the SBP in the morning (−10.0 mmHg, 95% CI: −16.5 to −3.5, *P* = 0.003), SBP at night (−10.2 mmHg, 95% CI: −16.1 to −4.3, *P* = 0.001), and DBP at night (−4.0 mmHg, 95% CI: −7.1 to −0.8, *P* = 0.014) all decreased significantly compared with the baseline. Also, the SBP in the morning (−7.6 mmHg, 95%CI: −14.1, −1.1, *P* = 0.023) decreased significantly at 3 weeks, and the SBP at night (−7.8 mmHg, 95%CI: −13.7, −2.0, *P* = 0.010) dropped at 2 weeks (Table 3). There was no statistically significant trend change in morning (*F* = 1.137, *P* = 0.352) and night diastolic (*F* = 0.344, *P* = 0.481) BPV and morning systolic BPV (*F* = 0.663, *P* = 0.930) over time during the intervention period, except for that night systolic BPV had a downward trend (*F* = 2.778, *P* = 0.016) and had decreased by 2.04 mmHg (95% CI: 0.84 to 3.23, *P* = 0.001) after intervention. Furthermore, the night diastolic BPV (−0.22 mmHg, 95% CI: −0.97 to 0.52, *P* = 0.553) and morning systolic BPV (−0.66 mmHg, 95% CI: −1.98 to 0.65, *P* = 0.319) also decreased, but the difference was not statistically significant (Figure 2). The data of morning and evening systolic BPV are seen in Table 4.

### 3.4. Safety

No serious adverse events occurred during the intervention. Other adverse events included the following: one patient had constipation at the first week, two patients developed fatigue, two patients developed dizziness and headache, and one patient had itching in the neck, back, and abdomen at the 3rd week.

## 4. Discussion

The Guidelines for Rational Use of Medicine in China suggest that it should be necessary to follow the principle of long-term efficacy and stability in regard to controlling blood pressure [17], which can prevent the occurrence of complications of cardiovascular and cerebrovascular diseases more effectively [18, 19]. Therefore, as a good measure to control the blood pressure in hypertensive patients, not only should it have the effect of lowering blood pressure but also, more importantly, maintain blood pressure stability.

The sodium content of traditional substitute salt is high, usually around 70% [7, 8], and some may be as low as 50% or even 25% [9, 20]. Previous salt-restricted studies have also shown that, in the case of relatively high sodium intake, the lower the sodium intake, the lower the blood pressure [21–23]. As an objective standard for measuring sodium and potassium intake, the 24-hour urine test has been widely used in scientific research. An international multicenter study showed that 24-h urine sodium/potassium was positively correlated with blood pressure, while 24-h urine potassium was negatively correlated with blood pressure [24]. A latest systematic review shows that the potassium intake of the Chinese population has been at a low level for the past 40 years, and the intake in all age groups is only half the recommended minimum or even lower [25]. The results of this study showed that, after 8 weeks of 18% substitute salt intervention, 24-hour urine potassium was significantly increased, while 24-hour sodium/potassium decreased, which indicates that this low-sodium salt has a more significant effect in increasing potassium intake. However, the effect of 24-hour urine sodium reduction is not obvious, which may be related to local eating habits of Chongqing people (preference for pickles, bean paste, and other high-salt foods).

Before intervention, we collected the clinical blood pressure of the patient at baseline, and the family self-measured blood pressure during 8 weeks of intervention. Compared to the baseline, we observed a decrease in blood pressure in the first week of the intervention and then a greater decrease in the second and three weeks. We have conducted a more detailed analysis of visit and home blood pressure in a separate article [26]. The SBP (SBP am: −10.0 mmHg and SBP pm: −10.2 mmHg) of patients seems to have dropped more than in previous studies (−7.52 mmHg) [5]. Also, there was a significant drop in blood pressure during the second week of the intervention, which appeared earlier than most previous studies [27–29]. In a prospective cohort study using 65% substitute salt, the blood pressure of hypertensive patients was not significantly reduced even after 18 months of intervention [29]. Based on these, we were concerned that the substitute salt (18% sodium content only) used in this study would lead to an increase in blood pressure variability due to lowering the blood pressure in a short time. However, in this study, we found that the patient's BPV did not increase significantly, and the variability of night systolic blood pressure even decreased during the intervention.

Previous studies have shown that the results of BPV are closely related to the number of blood pressure measurements [30, 31] and 6 blood pressure measurements are the minimum number of measurements that can better predict the occurrence of cardiovascular events [13]. In this study, blood pressure were measured 7 times a week to observe the BPV during the use of substitute salt, which can achieve a better response to BPV as well as better reflect the change cycle of blood pressure.

A study by Ozkayar et al. [32] showed that ambulatory blood pressure variability was positively correlated with dietary salt intake. In this study, BPV was the largest in the first week of intervention but decreased gradually during the intervention. The possible reasons for this decrease include reduction of salt use and ultra-low sodium chloride content, as well as effects of comprehensive factors such as regular medication, blood pressure management, and salt-sensitive population. However, as intervention continued, the body's own function was adjusted. When the blood pressure fell to the extent for the body to adapt, we observed that the variability of blood pressure gradually decreased.

There are still some limitations in this study. Firstly, the study was based on a phase I clinical trial that resulted in a limited sample size, and there are possibly sampling errors that make the results unstable. Furthermore, we used the BPV in the first week of intervention as the reference to observe the changes in BPV of patients during the intervention. If BPV decreased and stabilized in the first week of intervention due to the 18% substitute salt with gold sodium potassium ratio of 1 : 2, the effect of intervention may be underestimated. On the other hand, if BPV was significantly increased in the first week due to the influence of comprehensive factors compared with before the intervention, but as the intervention continued, the BPV gradually decreased after the body adapted to the intervention, the effect of this salt in reducing BPV would be overestimated.

## 5. Conclusions

Although 18% substitute salt has lower sodium content, its application in hypertensive patients did not cause an increase in blood pressure variability and even reduced the evening systolic blood pressure variability of the patients, which indicates that the 18% substitute salt has a good effect of controlling blood pressure. However, further long-term randomized controlled trials are still needed to verify this finding.

## Figures and Tables

**Table 1:** Demographic, clinical characteristics, and blood pressure at baseline.

Variables	All populations (*n* = 39)
Age, years (mean ± SD)	67.1 ± 7.9
Male (*n*, %)	20 (51.3)
BMI, kg/m^2^ (mean ± SD)	26.6 ± 3.6
Age of hypertension, years (mean ± SD)	11.0 ± 6.3
Complicated other diseases^*∗*^ (*n*, %)	19 (48.7)
Smoke (*n*, %)	4 (10.3)
Drink (*n*, %)	6 (18.2)
Exercises (*n*, %)	4 (10.3)
Follow-up SBP, mmHg (mean ± SD)	135.1 ± 16.8
Follow-up DBP, mmHg (mean ± SD)	72.6 ± 8.2
Morning SBP, mmHg (mean ± SD)	136.4 ± 18.7
Morning DBP, mmHg (mean ± SD)	76.3 ± 8.7
Evening SBP, mmHg (mean ± SD)	134.7 ± 18.1
Evening DBP, mmHg (mean ± SD)	74.3 ± 9.7

^*∗*^Ccomplicated other diseases include stroke, coronary heart disease, and diabetes mellitus.

**Table 2:** Changes of the 24-hour urine test before and after intervention.

Intervention	Sodium (mmol/24 h)	Potassium (mmol/24 h)	Sodium/potassium	Calcium (mmol/24 h)	Magnesium (mmol/24 h)	U-ALB/Cr (mg/g)
Before	151.86 ± 55.93	52.22 ± 19.30	3.05 (1.95∼4.36)	5.41 ± 2.40	4.35 ± 1.68	3.2 (1.9∼6.6)
After	138.05 ± 57.12	61.01 ± 17.26	2.39 (1.44∼3.28)	5.81 ± 3.23	4.77 ± 1.64	4.6 (2.8∼4.7)
Change	13.82 ± 69.23	−8.79 ± 22.76	0.62 (−0.19∼1.58)	−0.40 ± 2.62	−0.42 ± 1.43	−1.2 (−5.4∼0.0)
*t/z* value	1.246	−2.411	−3.265	−0.962	−1.839	−3.162
*P*	0.220	0.021	<0.001	0.342	0.074	0.002

**Table 3:** Changes in blood pressure from baseline during intervention, mean (95% CI)^*∗*^.

Week	SBP am		SBP pm		DBP am		DBP pm	
	Statistics	*P* ^*∗*^	Statistics	*P* ^*∗*^	Statistics	*P* ^*∗*^	Statistics	*P* ^*∗*^
Baseline	136.0 (130.2, 140.7)	−	133.8 (128.5, 139.0)	−	76.6 (74.2, 78.9)	−	74.4 (71.9, 77.0)	−
1	−3.1 (−10.0, 3.7)	0.365	−4.8 (−10.9, 1.4)	0.124	−0.6 (−3.9, 2.6)	0.705	−1.9 (−5.1, 1.4)	0.253
2	−6.2 (−12.7, 0.3)	0.060	−7.8 (−13.7, −2.0)	0.010	−1.7 (−4.9, 1.5)	0.284	−3.0 (−6.1, 0.2)	0.063
3	−7.6 (−14.1, −1.1)	0.023	−8.6 (−14.8, −2.4)	0.007	−2.0 (−5.3, 1.3)	0.232	−2.7 (−5.8, 0.4)	0.082
4	−8.9 (−15.4, −2.4)	0.008	−9.3 (−15.2, −3.4)	0.002	−2.2 (−5.6, 1.1)	0.189	−3.3 (−6.4, −0.3)	0.033
5	−9.9 (−16.2, −3.6)	0.003	−11.0 (−16.8, −5.1)	<0.001	−3.6 (−6.6, −0.6)	0.018	−3.9 (−7.0, −0.8)	0.014
6	−8.7 (−15.1, −2.3)	0.009	−10.1 (−15.8, −4.3)	0.001	−2.8 (−6.0, 0.3)	0.078	−3.4 (−6.5, −0.3)	0.031
7	−11.0 (−17.2, −4.7)	0.001	−11.9 (−17.8, −6.1)	<0.001	−3.3 (−6.4, −0.1)	0.041	−4.2 (−7.3, −1.1)	0.009
8	−10.0 (−16.5, −3.5)	0.003	−10.2 (−16.1, −4.3)	0.001	−2.8 (−6.1, 0.5)	0.097	−4.0 (−7.1, −0.8)	0.014

^*∗*^Adjusted for sex, age, body mass index, and reduction of antihypertensive drugs in the linear mixed model.

**Table 4:** Weekly changes in systolic blood pressure variability.

Week	SD of SBP am			SD of SBP pm		
	Mean, 95% CI	Mean difference^*∗*^, 95% CI	*P* ^*#*^	Mean, 95% CI	Mean difference^*∗*^, 95% CI	*P* ^*#*^
1	8.91 (7.55, 10.28)	−	−	9.86 (8.31, 11.42)	−	−
2	8.19 (6.82, 9.56)	−0.73 (−2.12, 0.67)	0.303	9.62 (7.98, 11.26)	−0.24 (−1.56, 1.08)	0.716
3	8.63 (7.28, 9.98)	−0.28 (−1.65, 1.08)	0.682	8.66 (7.08, 10.24)	−1.21 (−2.42, 0.01)	0.052
4	8.58 (7.33, 9.82)	−0.34 (−1.61, 0.93)	0.596	9.27 (7.47, 11.08)	−0.59 (−2.10, 0.92)	0.439
5	7.90 (6.47, 9.33)	−1.01 (−2.56, 0.43)	0.167	8.09 (6.54, 9.64)	−1.78 (−2.95, −0.59)	0.004
6	7.97 (6.76, 9.19)	−0.94 (−2.18, 0.30)	0.136	8.62 (7.02, 10.22)	−1.24 (−2.50, 0.02)	0.054
7	7.91 (6.61, 7.21)	−1.00 (−2.32, 0.31)	0.133	8.28 (6.76, 9.79)	−1.59 (−2.72, −0.46)	0.007
8	8.25 (6.95, 9.56)	−0.66 (−1.98, 0.65)	0.319	7.83 (6.26, 9.39)	−2.04 (−3.23, −0.84)	0.001

^*∗*^Mean difference compared with the first week. ^*∗*^Adjusted for sex, age, smoking, drinking, body mass index, and reduction of antihypertensive drugs in the linear mixed model.

## Data Availability

The datasets used and analyzed during the current study are available from the corresponding author on reasonable request.
